# Muscle quality correlates with hearing thresholds: a cross-sectional analysis

**DOI:** 10.3389/fragi.2025.1706350

**Published:** 2026-01-07

**Authors:** Young-Jee Jeon, Ji Ho Lee, Byung Chul Kang, Joong Keun Kwon

**Affiliations:** 1 Department of Family Medicine, Ulsan University Hospital, University of Ulsan College of Medicine, Ulsan, Republic of Korea; 2 Department of Occupational and Environmental Medicine, Ulsan University Hospital, University of Ulsan College of Medicine, Ulsan, Republic of Korea; 3 Department of Otorhinolaryngology, Ulsan University Hospital, University of Ulsan College of Medicine, Ulsan, Republic of Korea

**Keywords:** abdominal muscles, attenuation (absorption) coefficient, computed tomography, hearing, sarcopenia

## Abstract

**Introduction:**

Muscle health, including muscle volume, is an independent risk factor for physical disability and metabolic disorders. Although low muscle mass has been associated with hearing loss, the association between computed tomography (CT)-derived muscle quality and hearing has not been explored.

**Methods:**

This cross-sectional study examined associations between abdominal body composition and hearing thresholds in 7,774 adults (4,537 men and 3,237 women) aged ≥40 years undergoing routine health check-ups. Abdominopelvic CT and pure-tone audiometry were performed, and total abdominal muscle area (TAMA), normal-attenuation muscle area (NAMA), low-attenuation muscle area (LAMA), intermuscular adipose tissue (IMAT), visceral fat area (VFA), and subcutaneous fat area (SFA) were quantified from CT-based muscle quality maps. The best-ear pure-tone average (PTA, 1–4 kHz) was analyzed as the log_e_-transformed PTA. Multivariable linear regression models were fitted separately for men and women, adjusting for age, cardiovascular risk factors, and lifestyle variables.

**Results:**

The log_e_-transformed LAMA index was independently associated with hearing in both sexes. Each 10% increase in the log_e_-transformed LAMA index corresponded to an estimated 0.11 dB worsening of PTA in men and 0.14 dB worsening in women. In women, TAMA, NAMA, and VFA indices were associated with PTA; these associations were confined to women in their 50s and 60s in decade-stratified analyses. No abdominal body composition indices were significantly associated with hearing thresholds in any age group among men.

**Conclusions:**

These findings suggest that abdominal body composition is associated with age-related hearing loss (ARHL), particularly in midlife women, and highlight skeletal muscle health as a potential target for hearing preservation.

## Introduction

Age-related hearing loss (ARHL) is understood to be associated with multifactorial underlying risk factors beyond age, encompassing gender, ethnicity, environmental influences, lifestyle habits, cardiovascular risk factors, and genetic underpinnings (Bowl and Dawson, 2019).

Recent studies have highlighted immune system dysregulation as a key mechanism in ARHL. Inflammaging is an age-related low-grade sterile inflammatory state in tissues. In the auditory system, dysregulated tissue-resident macrophages primarily cause dysfunction in the strial microvasculature of the lateral cochlear wall. This leads to the secretion of pro-inflammatory cytokines and chemokines, potentially disrupting the blood–labyrinth barrier and damaging hair cells, neurons, and synaptic connections (Seicol et al., 2022; Parekh and Kaur, 2023).

Sarcopenia, a term initially coined to describe the age-related decrease in muscle mass, has been associated with an increased risk of falls, fractures, physical disability, and mortality. Studies have demonstrated a link between sarcopenia and systemic inflammation, along with sensory decline (Huang and He, 2025; Liu and Zhang, 2025). Several studies have indicated a negative correlation between muscle quantity and the prevalence of hearing loss (Lee et al., 2016; Kang et al., 2017; Park et al., 2022).

In recent years, the significance of muscle quality, in addition to muscle mass, has been recognized in the diagnostic process of sarcopenia (Cruz-Jentoft et al., 2019). Muscle quality is a relatively new concept, referring to both the function and structural characteristics of the muscle. The infiltration of adipose tissue into skeletal muscle, known as myosteatosis, has been demonstrated to exert a deleterious effect on muscle strength and function, thereby indicating poor muscle quality.

Myosteatosis has been demonstrated to be independently associated with metabolic disorders and elevated levels of chronic inflammation (Miljkovic et al., 2021). Given that myosteatosis appears earlier than muscle mass loss and considering the adverse effects of metabolic syndrome and chronic inflammation on ARHL, it may be reasonable to hypothesize that myosteatosis serves as an early marker for ARHL.

Computed tomography (CT) is considered the gold standard for quantitative and qualitative assessment of muscle because it can distinguish fat from muscle based on attenuation according to the tissue density and composition. Unlike muscle mass, muscle attenuation, defined by the Hounsfield unit (HU) on CT, reflects intramuscular fat infiltration and serves as a marker of muscle quality rather than quantity.

This study aimed to determine whether CT-derived muscle quality indices based on attenuation, such as low-attenuation muscle area (LAMA) or normal-attenuation muscle area (NAMA), are independently associated with hearing thresholds across sex and age groups.

## Materials and methods

### Study population

**FIGURE 1:**
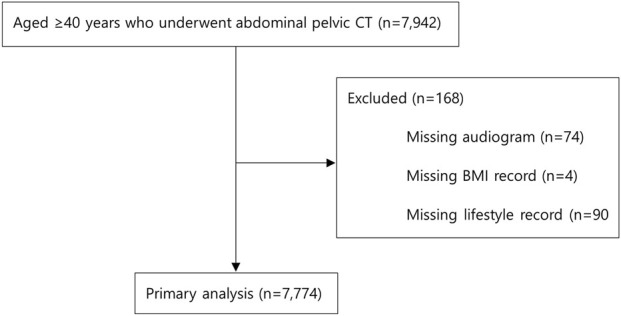
Overview of the study population.

Data were retrospectively collected from 7,942 participants aged ≥40 who underwent self-referral APCT and audiometry as part of routine check-ups at the Health Promotion Center, Ulsan University Hospital, between March 2014 and June 2019. For subjects who had repeated health examinations during the study period, data from the first examination were analyzed. Individuals were excluded if any of the clinical or lifestyle data, such as audiograms, body mass index (BMI), or self-reported questionnaires, were not available. Finally, a total of 7,774 persons (4,537 men and 3,237 women) were included in the analysis (Figure 1). Clinical and laboratory information was collected from the clinical data warehouse platform in conjunction with electronic medical records by the Big Data Center of Ulsan University Hospital.

### Clinical measurements

Clinical or lifestyle factors (e.g., hypertension, diabetes, dyslipidemia, history of heart disease, history of cerebrovascular accident, smoking status, alcohol consumption, physical activity, and occupational noise exposure) were collected from systemized self-report questionnaires administered to participants prior to their examination.

Hypertension was defined as being on antihypertensive medication or a systolic/diastolic blood pressure ≥140/90, according to the guideline of the Korean Society of Hypertension (Kim H.-L. et al., 2023). Diabetes was defined if subjects were taking anti-hyperglycemic drugs or had fasting blood glucose ≥126 mg/dL or HbA1c > 6.5% according to the clinical practice guidelines of diabetes (Hur et al., 2021). Dyslipidemia was defined as taking medication or having total cholesterol ≥240 mg/dL, triglycerides >200 mg/dL, HDL <40 mg/dL, or LDL >160 mg/dL, according to the Korean guidelines for the management of dyslipidemia (Jin et al., 2023). Smoking was classified into never smokers, former smokers, and current smokers. Drinking was categorized as never, moderate, or heavy, with heavy drinking defined as ≥ 14 drinks/week for men and ≥7 drinks/week for women, according to guidelines from the National Institute on Alcohol Abuse and Alcoholism (Chen et al., 2006). Physical activity was categorized into three levels based on the American College of Sports Medicine guidelines, with moderate to vigorous physical activity defined as ≥ 150 min of moderate exercise/week or ≥75 min of vigorous exercise/week (Garber et al., 2011). Noise-exposed occupations were categorized as engineers; agricultural, forestry, and fishery workers; skilled laborers; and military personnel, while the remaining occupations were defined as non-exposed.

### Hearing test

Pure-tone audiometry was performed in a sound-insulated booth using an audiometer (Grason-Stadler, Eden Prairie, Minnesota, United States) with supra-aural headphones. The pure-tone average (PTA) was calculated for each ear as the average hearing thresholds at 1, 2, 3, and 4 kHz measured in dB HL. The PTA of the best ear, which was reported to show a stronger association with physical performance than the worst ear, was selected for analysis (Kolasa et al., 2023). Sensitivity analysis was conducted using the binaural average of PTA.

### Image analysis of abdominopelvic CT

Computed tomography is considered the gold standard for the quantitative and qualitative assessment of muscle because it can distinguish fat from muscle based on attenuation, reflecting tissue density and composition. Intramyocellular lipid droplets, which could be considered low-attenuation muscle, and visible fat beneath muscle fascia and between muscle groups can be classified as myosteatosis on an APCT scan.

All APCT images were acquired using the SOMATOM Definition Flash system (Siemens Healthcare, Erlangen, Germany), as described previously (Lee et al., 2021). Enhanced images were obtained 80 s after contrast injection and reconstructed at a slice thickness of 3 mm.

Two consecutive axial APCT images were obtained at the inferior endplate of the L3 lumbar vertebra. The automatic segmentation was performed using deep learning-based software (Asan-J-Morphometry™, Asan Image Metrics, Seoul, Korea, http://datasharing.aim-aicro.com/morphometry). This software program was developed based on ImageJ (NIH, Bethesda, MD, United States) to demarcate the abdominal body compartment into the visceral fat area (VFA), subcutaneous fat area (SFA), and total abdominal muscle area (TAMA), encompassing all muscles within the field of target images (i.e., psoas, paraspinal, transversus abdominis, rectus abdominis, quadratus lumborum, and internal/external obliques). The software program used in this study demonstrated very high internal reliability when comparing post-contrast CT and MRI scans. Based on the TAMA criteria, the intraclass correlation coefficient (ICC) ranged from 0.85 to 0.99, and the within-subject coefficient of variation (WSCV) was approximately 8% (Park et al., 2019).

Second, TAMA was divided into three muscle components using the pixel-wise measurement of CT density: NAMA with +30 to +150 HU, LAMA with −29 to +29 HU, and intermuscular adipose tissue (IMAT) with −190 to −30 HU (Kim et al., 2020).

**FIGURE 2:**
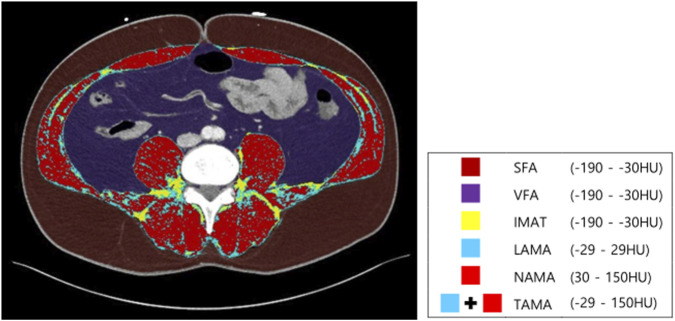
Abdominal body composition and muscle quality map generated from abdominopelvic computed tomography at the inferior endplate of L3 vertebra. HU, Hounsfield unit; SFA, subcutaneous fat area; VFA, visceral fat area; IMAT, intermuscular adipose tissue; LAMA, low-attenuation abdominal muscle area; NAMA, normal-attenuation muscle area; TAMA, total abdominal muscle area.

These values from the two consecutive axial images were averaged for each participant. A muscle quality map could be generated by combining these three muscle components (Figure 2). Poor-quality muscle, characterized by fatty degeneration (LAMA and IMAT), can be distinguished from healthy muscle, which is predominantly composed of NAMA.

The cross-sectional areas were adjusted by BMI based on the Foundation for the National Institutes of Health Sarcopenia Projection recommendation (Studenski et al., 2014). The following indices were referred to as the TAMA index (TAMAi) (TAMAi = TAMA [cm^2^]/BMI [kg/m^2^]), LAMA index (LAMAi) (LAMAi = LAMA [cm^2^]/BMI [kg/m^2^]), NAMA index (NAMAi) (NAMAi = NAMA [cm^2^]/BMI [kg/m^2^]), IMAT index (IMATi) (IMATi = IMATi [cm^2^]/BMI [kg/m^2^]), VFA index (VFAi) (VFAi = VFA [cm^2^]/BMI [kg/m^2^]), and SFA index (SFAi) (SFAi = SFA [cm^2^]/BMI [kg/m^2^]).

### Statistical analysis

Statistical analyses were performed separately for each sex as women are known to have better hearing than men. The clinical and lifestyle variables are presented as frequency with percentage for categorical variables and as mean with standard deviations for continuous variables.

Following an evaluation of the distribution of continuous variables, transformations were executed for variables exhibiting skewed deviations. For example, PTA and LAMAi were log_e_-transformed, and IMATi was square-root-transformed to normalize skewed distribution.

To ascertain the existence of a correlation between each abdominal body composition index and log_e_-transformed PTA, a series of linear regression model analyses were conducted.

The effects of abdominal body composition indices were then adjusted for a variety of clinical and lifestyle variables in multivariable regression analyses. We performed multivariable regression analyses using hierarchical regression. We placed age in block 1, clinical and lifestyle variables (e.g., hypertension, diabetes, dyslipidemia, history of heart disease or cerebrovascular accident, smoking status, alcohol consumption, physical activity, and occupational noise exposure) in block 2, and individual abdominal body composition indicators in block 3.

These correlations were re-examined by decade of age (i.e., 40 to 49, 50 to 59, 60 to 69, and 70 and older) to align with hormonal stages.

Statistical analyses were performed using SPSS version 24 for Windows (IBM Inc. Armonk, NY, United States). A *p-*value < 0.05 was considered statistically significant.

## Results

### Participant characteristics

The mean age of the subjects was 54.6 ± 7.8 years, and the mean PTA of the best ear was 16.0 ± 11.3 dB HL. Subject characteristics are listed in Table 1.

**TABLE 1:** Characteristics of the study participants.

Variable	Total (n = 7,774)	Male (n = 4,537)	Female (n = 3,237)
Age, year (range)	54.6 ± 7.8 (40–88)	54.3 ± 7.6 (40–84)	55.0 ± 8.1 (40–88)
PTA of best ear (dB HL)	16.0 ± 11.3	17.9 ± 12.2	13.3 ± 9.2
Hypertension (+)	2,274 (29.3%)	1,477 (32.6%)	797 (24.6%)
Diabetes (+)	904 (11.6%)	643 (14.2%)	261 (8.1%)
Dyslipidemia (+)	3,022 (38.9%)	2,030 (44.7%)	992 (30.6%)
History of heart disease (+)	239 (3.1%)	187 (4.1%)	52 (1.6%)
History of cerebrovascular accident (+)	57 (0.7%)	34 (0.7%)	23 (0.7%)
Smoking status			
Current smoker	1,565 (20.1%)	1,502 (33.1%)	63 (1.9%)
Former smoker	2,156 (27.7%)	2,105 (46.4%)	51 (1.6%)
Never	4,053 (52.1%)	930 (20.5%)	3,123 (96.5%)
Alcohol consumption			
Never drinking	3,082 (39.6%)	928 (20.5%)	2,154 (66.5%)
Moderate drinking	2,472 (31.8%)	1,731 (38.2%)	741 (22.9%)
Heavy drinking	2,220 (28.6%)	1,878 (41.4%)	342 (10.6%)
Physical activity			
Sedentary	2,897 (37.3%)	1,481 (32.6%)	1,416 (43.7%)
Light	3,134 (40.3%)	2,042 (45.0%)	1,092 (33.7%)
Moderate-to-vigorous	1,743 (22.4%)	1,014 (22.3%)	729 (22.5%)
Noise-exposed occupations (+)	1,950 (25.1%)	1,575 (34.7%)	375 (11.6%)
Abdominal body composition			
Total abdominal muscle area, cm^2^	135.3 ± 33.7	158.5 ± 22.8	102.8 ± 13.9
Normal-attenuation abdominal muscle area, cm^2^	110.0 ± 31.3	130.9 ± 21.8	80.6 ± 14.6
Low-attenuation muscle area, cm^2^	25.4 ± 10.4	27.6 ± 11.0	22.3 ± 8.6
Intermuscular adipose tissue, cm^2^	5.6 ± 4.0	5.5 ± 3.8	5.9 ± 4.2
Visceral fat area, cm^2^	106.3 ± 61.3	129.7 ± 61.8	73.7 ± 43.0
Subcutaneous fat area, cm^2^	142.2 ± 56.9	129.3 ± 51.6	160.3 ± 59.0
Abdominal body composition indices adjusted by body mass index			
Total abdominal muscle area index, cm^2^/(kg/m^2^)	5.6 ± 1.2	6.5 ± 0.7	4.5 ± 0.6
Normal-attenuation abdominal muscle area index, cm^2^/(kg/m^2^)	4.6 ± 1.2	5.4 ± 0.9	3.5 ± 0.7
Low-attenuation muscle area index, cm^2^/(kg/m^2^)	1.0 ± 0.4	1.1 ± 0.4	0.9 ± 0.3
Intermuscular adipose tissue index, cm^2^/(kg/m^2^)	0.2 ± 0.2	0.2 ± 0.1	0.2 ± 0.2
Visceral fat area index, cm^2^/(kg/m^2^)	4.3 ± 2.2	5.2 ± 2.2	3.1 ± 1.5
Subcutaneous fat area index, cm^2^/(kg/m^2^)	2.2 ± 0.8	1.8 ± 0.6	2.7 ± 0.8

Abbreviation: PTA, pure-tone averages of 1, 2, 3, and 4 kHz thresholds.

### Association between PTA of the best ear and abdominal body composition

In the univariable linear regression analysis, all abdominal body composition indices were associated with log_e_-transformed PTA in both sexes (Table 2).

**TABLE 2:** Univariate linear regression to determine the relationship with log_e_-transformed pure-tone averages of best ear (log_e_-transformed PTA). CI, confidence interval.

Gender	Male		Female	
Variable	Coefficient (95% CI)	*p*	Coefficient (95% CI)	*p*
Total abdominal muscle area index, cm^2^/(kg/m^2^)	−0.133 (−0.159 to −0.108)	0.000	−0.246 (−0.278 to −0.214)	0.000
Normal-attenuation muscle area index, cm^2^/(kg/m^2^)	−0.147 (−0.168 to −0.126)	0.000	−0.267 (−0.293 to −0.241)	0.000
Log_e_ (low-attenuation muscle area index), cm^2^/(kg/m^2^)	0.314 (0.261–0.368)	0.000	0.523 (0.462–0.584)	0.000
Root square (intermuscular adipose tissue index), cm^2^/(kg/m^2^)	0.322 (0.200–0.444)	0.000	0.725 (0.601–0.848)	0.000
Visceral fat area index, cm^2^/(kg/m^2^)	0.014 (0.005–0.022)	0.002	0.090 (0.078–0.103)	0.000
Subcutaneous fat area index, cm^2^/(kg/m^2^)	−0.085 (−0.118 to −0.052)	0.000	0.082 (0.058–0.107)	0.000

After adjusting for clinical and lifestyle variables, only log_e_-transformed LAMAi was associated with log_e_-transformed PTA in men [unstandardized β (95% confidence interval [CI]) = 0.069 (0.020–0.118), *p* = 0.006], indicating a 0.11-dB worsening in PTA for a 10% increase in log_e_-transformed LAMAi.

In women, log_e_-transformed LAMAi [unstandardized β (95% CI) = 0.109 (0.051–0.167), *p* = 0.000], TAMAi [unstandardized β (95% CI) = −0.036 (−0.067 to −0.006), *p* = 0.019], NAMAi [unstandardized β (95% CI) = −0.052 (−0.079 to −0.025), *p* = 0.000], and VFAi [unstandardized β (95% CI) = 0.018 (0.006–0.029), *p* = 0.003] were associated with log_e_-transformed PTA (Table 3). These results correspond to a 0.14 dB change in PTA for a 10% difference in log_e_-transformed LAMAi, a −0.05 dB change in PTA for a 10% difference in TAMAi, a −0.07 dB change in PTA for a 10% difference in NAMAi, and a 0.02 dB change in PTA for a 10% difference in VFAi (Table 3).

**TABLE 3:** Multivariable linear regression to determine the relationship with log_e_-transformed pure-tone averages of best ear (log_e_-transformed PTA) after adjustment of clinical and lifestyle factors. CI, confidence interval.

Gender	Male				Female			
Variable	Coefficient (95% CI)	*p*	VIF	Adjusted *R* ^2^	Coefficient (95% CI)	*p*	VIF	Adjusted *R* ^2^
Total abdominal muscle area index, cm^2^/(kg/m^2^)	−0.004 (−0.027 to 0.019)	0.733	1.103	0.262	−0.036 (−0.066 to −0.005)	0.023	1.216	0.311
Normal-attenuation muscle area index, cm^2^/(kg/m^2^)	−0.017 (−0.036 to 0.003)	0.097	1.158	0.262	−0.052 (−0.079 to −0.025)	0.000	1.384	0.313
Log_e_ (low-attenuation muscle area index), cm^2^/(kg/m^2^)	0.069 (0.020–0.118)	0.006	1.114	0.263	0.109 (0.051–0.167)	0.000	1.220	0.313
Root square (intermuscular adipose tissue index), cm2/(kg/m2)	−0.004 (-0.111 to 0.103)	0.946	1.307	0.262	0.079 (−0.032–0.189)	0.162	1.183	0.310
Visceral fat area index, cm^2^/(kg/m^2^)	−0.003 (−0.010 to 0.005)	0.500	1.054	0.262	0.018 (0.006–0.029)	0.003	1.188	0.312
Subcutaneous fat area index, cm^2^/(kg/m^2^)	−0.023 (−0.051 to 0.006)	0.119	1.016	0.262	0.018 (−0.003–0.039)	0.098	1.034	0.311

Multivariable linear regression stratified by 10-year age groups showed that TAMAi, NAMAi, log_e_-transformed LAMAi, and VFAi were associated with log_e_-transformed PTA in women in their 50s. NAMAi and log_e_-transformed LAMAi remained associated with log_e_-transformed PTA in women in their 60s (Table 4).

**TABLE 4:** Multiple linear regression analysis of association between log_e_-transformed pure-tone averages of best ear (log_e_-transformed PTA) and abdominal body composition indices by 10-year age group after adjustment of clinical and lifestyle factors in female individuals.

Sex	Female			
Age range (years) (n)	40–49 (859)	50–59 (1,517)	60–69 (690)	≥ 70 (171)
PTA of best ear (dB HL)	8.6 ± 4.6	12.2 ± 7.0	18.0 ± 10.0	28.5 ± 14.4
Abdominal body composition index	Unstandardized coefficient (95% confidence interval), *p*-value			
Total abdominal muscle area index, cm^2^/(kg/m^2^)	0.001 (−0.045 to 0.048), *p* = 0.959 VIF = 1.002/adj *R* ^2^ = 0.036	−0.057 (−0.102 to −0.011), *p* = 0.015 VIF = 1.057/adj *R* ^2^ = 0.052	−0.056 (−0.132 to 0.021), *p* = 0.152 VIF = 1.017/adj *R* ^2^ = 0.040	−0.058 (−0.205 to 0.089), *p* = 0.437 VIF = 1.040/adj *R* ^2^ = 0.103
Normal-attenuation muscle area index, cm^2^/(kg/m^2^)	−0.013 (−0.055 to 0.029), *p* = 0.549 VIF = 1.005/adj *R* ^2^ = 0.036	−0.064 (−0.105 to −0.024), *p* = 0.002 VIF = 1.086/adj *R* ^2^ = 0.055	−0.076 (−0.143 to −0.010), *p* = 0.025 VIF = 1.040/adj *R* ^2^ = 0.045	−0.088 (−0.215 to 0.038), *p* = 0.171 VIF = 1.093/adj *R* ^2^ = 0.110
Log_e_ (low-attenuation muscle area index), cm^2^/(kg/m^2^)	0.094 (−0.004 to 0.191), *p* = 0.060 VIF = 1.016/adj *R* ^2^ = 0.040	0.088 (0.003–0.173), *p* = 0.043 VIF = 1.033/adj *R* ^2^ = 0.051	0.152 (0.012–0.293), *p* = 0.034 VIF = 1.033/adj *R* ^2^ = 0.044	0.218 (−0.068 to 0.504), *p* = 0.134 VIF = 1.062/adj *R* ^2^ = 0.112
Root square (intermuscular adipose tissue index), cm^2^/(kg/m^2^)	0.093 (−0.102 to 0.287), *p* = 0.349 VIF = 1.001/adj *R* ^2^ = 0.037	0.061 (−0.102 to 0.225), *p* = 0.462 VIF = 1.022/adj *R* ^2^ = 0.049	0.097 (−0.174 to 0.367), *p* = 0.483 VIF = 1.001/adj *R* ^2^ = 0.038	0.071 (−0.347 to 0.490), *p* = 0.737 VIF = 1.029/adj *R* ^2^ = 0.101
Visceral fat area index, cm^2^/(kg/m^2^)	0.016 (−0.006 to 0.037), *p* = 0.147 VIF = 1.008/adj *R* ^2^ = 0.038	0.019 (0.001–0.037), *p* = 0.035 VIF = 1.053/adj *R* ^2^ = 0.051	0.022 (−0.004 to 0.047), *p* = 0.093 VIF = 1.001/adj *R* ^2^ = 0.041	0.025 (−0.017 to 0.067), *p* = 0.235 VIF = 1.010/adj *R* ^2^ = 0.108
Subcutaneous fat area index, cm^2^/(kg/m^2^)	0.014 (−0.022 to 0.049), *p* = 0.448 VIF = 1.002/adj *R* ^2^ = 0.037	0.024 (−0.008 to 0.056), *p* = 0.136 VIF = 1.021/adj *R* ^2^ = 0.050	0.013 (−0.033 to 0.060), *p* = 0.578 VIF = 1.005/adj *R* ^2^ = 0.037	0.032 (−0.063 to 0.128), *p* = 0.505 VIF = 1.007/adj *R* ^2^ = 0.103

Abbreviation: PTA, pure-tone averages of 1, 2, 3, and 4 kHz thresholds; Adj *R* ^2^, adjusted *R* ^2^.

None of the abdominal body composition indices of the male participants, including log_e_-transformed LAMAi, were significantly associated with log_e_-transformed PTA in any age group when stratified by decade (Supplementary Table S1).

In the sensitivity analysis, when the binaural average PTA was used as the hearing result instead of PTA of the best ear, only NAMAi, rather than log_e_-transformed LAMAi, was associated with the log_e_-transformed PTA in men. In women, however, SFAi, along with TAMAi, NAMAi, log_e_-transformed LAMAi, and VFAi, was associated with the log_e_-transformed binaural PTA (Supplementary Table S2). When data were stratified by 10-year age groups, TAMAi and NAMAi were associated with the log_e_-transformed binaural PTA in women in their 50s. NAMAi and log_e_-transformed LAMAi were associated with the log_e_-transformed binaural PTA in women in their 60s (Supplementary Table S3). In men, only SFAi was correlated with the log_e_-transformed binaural PTA (Supplementary Table S4).

## Discussion

This study provides evidence of a significant association between the abdominal body composition and hearing level.

LAMA, a key marker of poor muscle quality, was significantly associated with the best-ear hearing threshold in both sexes. The association of TAMA, NAMA, and VFA with best-ear hearing was only found in women. When analyzed by decade, the associations of TAMA, NAMA, and VFA with the best-ear hearing level were significant in women in their 50s, while NAMA and LAMA, which represent skeletal muscle quality, remained associated with best-ear hearing in women in their 60s.

When we repeated the analyses using the binaural PTA as a sensitivity analysis, the overall pattern was largely preserved: TAMAi and NAMAi remained associated with the binaural PTA in women in their 50s, while NAMAi and log_e_-transformed LAMAi again showed significant associations in their 60s, suggesting that the association between abdominal muscle quality and hearing is robust to the definition of the audiometric outcome.

For men, our data did not consistently show an association between abdominal body composition and hearing.

Studies examining the association between indicators of sarcopenia—such as handgrip strength, physical function, gait speed, and physical performance—and hearing have reported that the risk of hearing loss increases as physical function decreases (Ho et al., 2022).

Fewer studies have been conducted on the relationship between skeletal muscle mass and hearing. These studies have indicated that lower muscle mass is associated with an increased risk of hearing loss, with this effect being more pronounced in women (Lee et al., 2016; Kang et al., 2017; Park et al., 2022). The assessment of skeletal muscle mass was performed using dual-energy x-ray absorptiometry (DXA) or bioelectrical impedance analysis (BIA) in these studies. Given that DXA and BIA cannot assess intramyocellular lipid, the question of whether muscle mass measured using these methods better reflects TAMA or NAMA on CT scans remains unclear. In this study, NAMA was found to be more consistently associated with hearing level than TAMA.

West et al. (2019) reported that low-attenuation abdominal muscle, but not muscle mass, was associated with reduced aerobic fitness, as measured by poor oxygen uptake at peak exercise. Additionally, it has been reported that subjects older than 50 years who exhibited poor oxygen uptake during peak exercise had worse hearing (Alessio et al., 2002). These findings are consistent with our results that LAMA showed the strongest correlation coefficient with hearing thresholds.

Myosteatosis is associated with chronic inflammation, and this inflammation may contribute to the conceptual framework of ARHL by causing damage to the stria vascularis, cochlear hair cells, spiral ganglion, and cochlear nucleus (Miljkovic et al., 2021; Parekh and Kaur, 2023).

A substantial body of research has indicated a correlation of an elevated risk of metabolic syndrome with abdominal body composition, including lower muscle mass, LAMA, and visceral fat (Son et al., 2017; Lee et al., 2021; Abildgaard et al., 2018). Metabolic syndrome has also been demonstrated to be closely associated with ARHL. The present study corroborated the independent association of each component of abdominal body composition, including TAMA, NAMA, LAMA, and VFA, with hearing level after adjustment for both clinical and lifestyle metabolic risk factors.

Furthermore, the correlation between each abdominal body composition index and metabolic syndrome or hearing level exhibited divergent patterns depending on gender. For instance, the association between NAMA and metabolic syndrome has been documented in both sexes (Lee et al., 2021). However, the correlation between NAMA and hearing level was only significant in the female population. The association between LAMA and metabolic syndrome has been demonstrated in male subjects only (Lee et al., 2021). In contrast, a statistically significant association between LAMA and hearing level was identified in both sexes. The VFA exhibited a common characteristic, demonstrating a significant association with hearing levels and metabolic syndrome exclusively in women (Schorr et al., 2018; Lee et al., 2021). This discrepancy suggests the possibility of analogous yet discrete mechanisms underlying the correlation between abdominal body composition and hearing thresholds, potentially different from those associated with metabolic syndrome.

When analyzed by decade of age, the association between the abdominal body composition and hearing level was primarily observed in women in their 50s and 60s.

Menopause is accompanied by a redistribution of adipose tissue toward central and visceral depots, along with changes in ectopic fat accumulation, and these changes have been associated with lower circulating estrogen levels (Abildgaard et al., 2018). Estrogen has been implicated in the maintenance of skeletal muscle mass and function, although the detailed mechanisms remain incompletely understood (Hosoi et al., 2024). Experimental and clinical data also suggest that female sex hormones exert protective effects on the auditory system and may delay age-related or noise-induced hearing loss (Shuster et al., 2019). Thus, the abrupt decrease in estrogen during menopause could adversely influence muscle quality, visceral adiposity, and hearing, which offers a biologically plausible explanation—although not proof—for the stronger association between abdominal body composition and hearing level observed in women in the perimenopausal transition.

Androgens are well known for their anabolic effects on skeletal muscle and may contribute to the maintenance of muscle mass with aging (Gallo-Soljancic et al., 2025). In contrast, evidence regarding androgens and hearing is more limited and mixed; animal and human studies indicate that higher prenatal androgen exposure can masculinize and sometimes weaken cochlear function, but the role of circulating testosterone in adult hearing remains unclear (McFadden, 2009; Yang et al., 2023). The absence of a clear association between the abdominal body composition and hearing level in men in our data might, therefore, reflect both the more gradual age-related decrease in testosterone and the lack of a well-established protective effect of androgens on hearing.

Intermuscular fat, another well-documented marker of myosteatosis, did not exhibit a correlation with the hearing threshold. A similar set of findings has also been reported regarding the association with metabolic syndrome. Notwithstanding the significant correlation between insulin resistance and LAMA after adjusting for clinical and laboratory factors, the correlation between insulin resistance and IMAT was not significant (Kim M. J. et al., 2023). This outcome may be influenced by the small proportion of IMAT within the total abdominal muscle on CT images.

The association between abdominal body composition and hearing thresholds was not observed in subjects aged 70 years and older, suggesting that other age-related factors may have a greater impact on hearing in this age group.

Our study has several limitations. First, this is a cross-sectional study, so it is not possible to determine whether changes in abdominal composition directly contribute to age-related hearing loss or are merely a biomarker. Our findings suggest an association rather than causation, which requires longitudinal validation. Second, potential selection bias and limited generalizability could exist because the participants were self-referred for health check-ups rather than randomly sampled. Third, no information was available regarding actual muscle performance. Fourth, the audiogram only assessed 1–4 kHz, and the low- and high-frequency audiograms were missing due to limitations in the check-up protocol. Fifth, important confounders were missing from the routine check-up questionnaire, such as otologic diseases, use of ototoxic medications, and duration of noise exposure, all of which may impact the results. Sixth, enhanced CT was used to analyze abdominal body composition. Post-contrast HU values differ from the non-contrast scans, which may bias the NAMA and LAMA thresholds. Recently, a growing number of studies utilizing enhanced CT images have been published. Since muscle HU values can vary depending on the contrast phase, studies on muscle quality using enhanced CT require standardization (Kim et al., 2021).

Nevertheless, the strength of this study is that it is the first to examine the correlation between muscle quality and hearing level using muscle quality maps from CT in a large sample of subjects from the general population.

## Conclusion

Reduced muscle quality was independently associated with higher hearing thresholds; longitudinal and interventional studies are needed to clarify causality. Future research should focus on longitudinal studies to confirm causality and explore the mechanisms underlying the observed association.

## Data Availability

The raw data supporting the conclusions of this article will be made available by the authors, without undue reservation.
