# Correction: Muscle quality correlates with hearing thresholds: a cross-sectional analysis

**DOI:** 10.3389/fragi.2026.1823512

**Published:** 2026-03-18

**Authors:** Young-Jee Jeon, Ji Ho Lee, Byung Chul Kang, Joong Keun Kwon

**Affiliations:** 1 Department of Family Medicine, Ulsan University Hospital, University of Ulsan College of Medicine, Ulsan, Republic of Korea; 2 Department of Occupational and Environmental Medicine, Ulsan University Hospital, University of Ulsan College of Medicine, Ulsan, Republic of Korea; 3 Department of Otorhinolaryngology, Ulsan University Hospital, University of Ulsan College of Medicine, Ulsan, Republic of Korea

**Keywords:** abdominal muscles, attenuation (absorption) coefficient, computed tomography, hearing, sarcopenia

**Affiliation 1** “Department of Family Medicine, Ulsan University Hospital, University of Ulsan College of Medicine, Ulsan, Republic of Korea” was erroneously given as “Department of Family Medicine, Ulsan University Hospital, Ulsan, Republic of Korea”.

**Affiliation 2** “Department of Occupational and Environmental Medicine, Ulsan University Hospital, University of Ulsan College of Medicine, Ulsan, Republic of Korea” was erroneously given as “Department of Occupational and Environmental Medicine, Ulsan University Hospital, Ulsan, Republic of Korea”.

**Affiliation 3** “Department of Otorhinolaryngology, Ulsan University Hospital, University of Ulsan College of Medicine, Ulsan, Republic of Korea” was erroneously given as “Department of Otorhinolaryngology, Ulsan University Hospital, Ulsan, Republic of Korea”.

The author contributions statement was erroneously given as “Y-JJ: Writing – original draft, Conceptualization, Methodology, Project administration. JL: Writing – original draft, Data curation. BK: Investigation, Validation, Writing – review and editing. JK: Funding acquisition, Investigation, Supervision, Validation, Writing – review and editing”. The correct author contributions statement is “Y-JJ: Writing – original draft, Writing – review and editing, Conceptualization, Methodology, Project administration. JL: Writing – original draft, Writing – review and editing, Data curation. BK: Investigation, Validation, Writing – original draft, Writing – review and editing. JK: Funding acquisition, Investigation, Supervision, Validation, Writing – original draft, Writing – review and editing”

The original article has been updated

