# Supplementary material for: Muscle quality correlates with hearing thresholds: a cross-sectional analysis
Source: Front Aging. 2026 Jan 7;6:1706350. doi: 10.3389/fragi.2025.1706350 (PMC12819218; doi:10.3389/fragi.2025.1706350)
Supplement: Supplementary file 1 [file Table1.docx]

Table 1. Multiple linear regression analysis of association between log_e_-transformed pure tone averages of best ear (log_e_-transformed PTA) and abdominal body composition indices by 10-year age group after adjustment of clinical and lifestyle factors in males.

| **Sex** | **Male** | | | |
| --- | --- | --- | --- | --- |
| Age range (years) (n) | 40 ~ 49 (1,245) | 50 ~ 59 (2,232) | 60 ~ 69 (908) | >= 70 (152) |
| PTA of best ear (dB HL) | 11.9 ± 7.6 | 17.4 ± 10.7 | 24.4 ± 13.5 | 36.3 ± 17.6 |
| Abdominal body composition index | Unstandardized coefficient (95% Confidence interval), *p* value | | | |
| Total abdominal muscle area index, cm^2^/(kg/m^2^) | 0.008 (-0.033 to 0.049)  *p* = 0.710  VIF=1.005 / adj R^2^=0.052 | 0.001 (-0.034 to 0.035)  *p* = 0.974  VIF=1.017 / adj R^2^=0.065 | -0.013 (-0.067 to 0.040)  *p* = 0.624  VIF=1.054 / adj R^2^=0.068 | -0.051 (-0.169 to 0.067)  *p* = 0.393  VIF=1.042 / adj R^2^=0.075 |
| Normal attenuation muscle area index, cm^2^/(kg/m^2^) | -0.010 (-0.045 to 0.025)  *p* = 0.418  VIF=1.015 / adj R^2^=0.053 | -0.014 (-0.043 to 0.016)  *p* = 0.364  VIF=1.027 / adj R^2^=0.065 | -0.016 (-0.060 to 0.028)  *p* = 0.480  VIF=1.046 / adj R^2^=0.068 | -0.066 (-0.161 to 0.029)  *p* = 0.170  VIF=1.036 / adj R^2^=0.082 |
| Log_e_ (Low attenuation muscle area index), cm^2^/(kg/m^2^) | 0.090 (-0.001 to 0.180)  *p* = 0.051  VIF=1.059 / adj R^2^=0.056 | 0.065 (-0.008 to 0.138)  *p* = 0.079  VIF=1.034 / adj R^2^=0.066 | 0.040 (-0.067 to 0.148)  *p* = 0.460  VIF=1.022 / adj R^2^=0.068 | 0.142 (-0.090 to 0.375)  *p* = 0.228  VIF=1.011 / adj R^2^=0.079 |
| Root square (Intermuscular fat area index), cm2/(kg/m2) | -0.081 (-0.282 to 0.120)  *p* = 0.427  VIF=1.008 / adj R^2^=0.053 | -0.045 (-0.205 to 0.116)  *p* = 0.585  VIF=1.011 / adj R^2^=0.065 | 0.116 (-0.111 to 0.344)  *p* = 0.316  VIF=1.040 / adj R^2^=0.069 | 0.344 (-0.131 to 0.819)  *p* = 0.154  VIF=1.018 / adj R^2^=0.081 |
| Visceral fat area index, cm2/(kg/m2) | 0.000 (-0.015 to 0.015)  *p* = 0.971  VIF=1.056 / adj R^2^=0.052 | -0.003 (-0.014 to 0.008)  *p* = 0.627  VIF=1.043 / adj R^2^=0.065 | -0.003 (-0.018 to 0.011)  *p* = 0.642  VIF=1.005 / adj R^2^=0.068 | -0.009 (-0.039 to 0.021)  *p* = 0.538  VIF=1.000 / adj R^2^=0.074 |
| Subcutaneous fat area index, cm^2^/(kg/m^2^) | -0.036 (-0.084 to 0.013)  *p* = 0.148  VIF=1.020 / adj R^2^=0.054 | -0.020 (-0.064 to 0.023)  *p* = 0.363  VIF=1.014 / adj R^2^=0.065 | -0.042 (-0.107 to 0.023)  *p* = 0.203  VIF=1.037 / adj R^2^=0.069 | 0.087 (-0.074 to 0.248)  *p* = 0.288  VIF=1.029 / adj R^2^=0.076 |

Abbreviation: PTA, pure-tone averages of 1kHz, 2kHz, 3kHz, 4kHz thresholds; Adj R^2^, adjusted R^2^

Table 2. Multivariable linear regression to determine the relationship with log_e_-transformed binaural average of PTA (log_e_-transformed PTA) after adjustment of clinical and lifestyle factors as sensitivity analysis. *CI*, Confidence interval

| Gender | Male | | | | Female | | | |
| --- | --- | --- | --- | --- | --- | --- | --- | --- |
| Variables | Coefficient (95% CI) | *p* | VIF | Adjusted R^2^ | Coefficient (95% CI) | *p* | VIF | Adjusted R^2^ |
| Total abdominal muscle area index, cm^2^/(kg/m^2^) | 0.005 (-0.018 to 0.027) | 0.679 | 1.108 | 0.257 | -0.035 (-0.065 to -0.004) | 0.026 | 1.129 | 0.299 |
| Normal attenuation muscle area index, cm^2^/(kg/m^2^) | -0.008 (-0.027 to 0.011) | 0.408 | 1.165 | 0.257 | -0.047 (-0.075 to -0.025) | 0.020 | 1.392 | 0.300 |
| Log_e_ (Low attenuation muscle area index), cm^2^/(kg/m^2^) | 0.056 (0.009 to 0.1045) | 0.021 | 1.118 | 0.258 | 0095 (0.036 to 0.154) | 0.002 | 1.226 | 0.300 |
| Root square (Intermuscular adipose tissue index), cm2/(kg/m2) | -0.024 (-0.128 to 0.080) | 0.650 | 1.037 | 0.257 | 0.071 (-0.041 to 0.182) | 0.214 | 1.118 | 0.298 |
| Visceral fat area index, cm^2^/(kg/m^2^) | -0.004 (-0.011 to 0.004) | 0.318 | 1.072 | 0.257 | 0.015 (0.003 to 0.027) | 0.015 | 1.236 | 0.299 |
| Subcutaneous fat area index, cm^2^/(kg/m^2^) | -0.022 (-0.050 to 0.005) | 0.116 | 1.017 | 0.258 | 0.021 (0.000 to 0.042) | 0.049 | 1.035 | 0.299 |

Abbreviation: PTA, pure-tone averages of 1kHz, 2kHz, 3kHz, 4kHz thresholds

Table 3. Multiple linear regression analysis of association between log_e_-transformed binaural average of PTA (log_e_-transformed PTA) and abdominal body composition indices by 10-year age group after adjustment of clinical and lifestyle factors in females.

| **Sex** | **Female** | | | |
| --- | --- | --- | --- | --- |
| Age range (years) (n) | 40 ~ 49 (859) | 50 ~ 59 (1,517) | 60 ~ 69 (690) | >= 70 (171) |
| PTA of both ear (dB HL) | 10.1 ± 5.8 | 14.4 ± 8.4 | 20.7 ± 11.3 | 31.9 ± 15.5 |
| Abdominal body composition index | Unstandardized coefficient (95% Confidence interval), *p* value | | | |
| Total abdominal muscle area index, cm^2^/(kg/m^2^) | -0.008 (-0.057 to 0.041)  *p* = 0745  VIF=1.002 / adj R^2^=0.038 | -0.051 (-0.098 to -0.005)  *p* = 0.031  VIF=1.059 / adj R^2^=0.048 | -0.059 (-0.133 to 0.015)  *p* = 0.152  VIF=1.017 / adj R^2^=0.034 | -0.053 (-0.192 to 0.087)  *p* = 0.457  VIF=1.040 / adj R^2^=0.08 |
| Normal attenuation muscle area index, cm^2^/(kg/m^2^) | 0.018 (-0.062 to 0.026)  *p* = 0.418  VIF=1.005 / adj R^2^=0.039 | -0.056 (-0.097 to -0.015)  *p* = 0.007  VIF=1.093 / adj R^2^=0.050 | -0.080(-0.145 to -0.015)  *p* = 0.015  VIF=1.040 / adj R^2^=0.039 | -0.080 (-0.200 to 0.040)  *p* = 0.190  VIF=1.040 / adj R^2^=0.092 |
| Log_e_ (Low attenuation muscle area index), cm^2^/(kg/m^2^) | 0.078 (-0.024 to 0.180)  *p* = 0.135  VIF=1.016 / adj R^2^=040 | 0.073 (-0.014 to 0.159)  *p* = 0.100  VIF=1.041 / adj R^2^=0.047 | 0.158(0.021 to 0.294)  *p* = 0.023  VIF=1.033 / adj R^2^=0.038 | 0.184 (-0.087 to 0.455)  *p* = 0.183  VIF=1.062 / adj R^2^=0.092 |
| Root square (Intermuscular adipose tissue index), cm2/(kg/m2) | 0.055 (-0.148 to 0.259)  *p* = 0.593  VIF=1.001 / adj R^2^=0.038 | 0.066 (-0.100 to 0.232)  *p* = 0.438  VIF=1.0022 / adj R^2^=0.046 | 0.118 (-0.144 to 0.379)  *p* = 0.376  VIF=1.018 / adj R^2^=0.032 | 0.085 (-0.311 to 0.480)  *p* = 0.673  VIF=1.029 / adj R^2^=0.084 |
| Visceral fat area index, cm2/(kg/m2) | 0.018 (-0.004 to 0.041)  *p* = 0.113  VIF=1.008 / adj R^2^=0.041 | 0.015 (-0.003 to 0.034)  *p* = 0.099  VIF=1.091 / adj R^2^=0.047 | 0.016(-0.008 to 0.041)  *p* = 0.191  VIF=1.010 / adj R^2^=0.033 | 0.026 (-0.014 to 0.065)  *p* = 0.200  VIF=1.010 / adj R^2^=0.092 |
| Subcutaneous fat area index, cm^2^/(kg/m^2^) | 0.015 (-0.023 to 0.052)  *p* = 0.444  VIF=1.002 / adj R^2^=0.039 | 0.029 (-0.003 to 0.062)  *p* = 0.080  VIF=1.022 / adj R^2^=0.047 | 0.014 (-0.032 to 0.059)  *p* = 0.553  VIF=1.005 / adj R^2^=0.032 | 0.054 (-0.036 to 0.144)  *p* = 0.238  VIF=1.007 / adj R^2^=0.090 |

Abbreviation: PTA, pure-tone averages of 1kHz, 2kHz, 3kHz, 4kHz thresholds; Adj R^2^, adjusted R^2^

Table 4. Multiple linear regression analysis of association between log_e_-transformed binaural average of PTA (log_e_-transformed PTA) and abdominal body composition indices by 10-year age group after adjustment of clinical and lifestyle factors in males.

| **Sex** | **Male** | | | |
| --- | --- | --- | --- | --- |
| Age range (years) (n) | 40 ~ 49 (1,245) | 50 ~ 59 (2,232) | 60 ~ 69 (908) | >= 70 (152) |
| PTA of both ear (dB HL) | 14.3 ± 8.8 | 20.6 ± 17.7 | 28.0 ± 14.2 | 40.5 ± 17.8 |
| Abdominal body composition index | Unstandardized coefficient (95% Confidence interval), *p* value | | | |
| Total abdominal muscle area index, cm^2^/(kg/m^2^) | 0.022 (-0.019 to 0.063)  *p* = 0.299  VIF=1.005 / adj R^2^=0.048 | 0.009 (-0.024 to 0.042)  *p* = 0.579  VIF=1.017 / adj R^2^=0.064 | -0.013 (-0.064 to 0.037)  *p* = 0.604  VIF=1.054 / adj R^2^=0.060 | -0.082 (-0.192 to 0.029)  *p* = 0.146  VIF=1.042 / adj R^2^=0.076 |
| Normal attenuation muscle area index, cm^2^/(kg/m^2^) | 0.002 (-0.033 to 0.038)  *p* = 0.895  VIF=1.015 / adj R^2^=0.047 | -0.007(-0.035 to 0.022)  *p* = 0.654  VIF=1.027 / adj R^2^=0.064 | -0.017 (-0.059 to 0.025)  *p* = 0.422  VIF=1.045 / adj R^2^=0.061 | -0.081 (-0.171 to 0.008)  *p* = 0.074  VIF=1.047 / adj R^2^=0.082 |
| Log_e_ (Low attenuation muscle area index), cm^2^/(kg/m^2^) | 0.076 (-0.015 to 0.166)  *p* = 0.101  VIF=1.059 / adj R^2^=0.049 | 0.061 (-0.008 to 0.131)  *p* = 0.085  VIF=1.034 / adj R^2^=0.065 | 0.040 (-0.061 to 0.141)  *p* = 0.432  VIF=1.012 / adj R^2^=0.060 | 0.120 (-0.099 to 0.340)  *p* = 0.280  VIF=1.011 / adj R^2^=0.070 |
| Root square (Intermuscular fat area index), cm2/(kg/m2) | -0.129 (-0.331 to 0.073)  *p* = 0.210  VIF=1.008 / adj R^2^=0.048 | -0.055 (-0.209 to 0.099)  *p* = 0.482  VIF=1.011 / adj R^2^=0.064 | 0.122(-0.092 to 0.336)  *p* = 0.265  VIF=1.009 / adj R^2^=0.061 | 0.360 (-0.088 to 0.809)  *p* = 0.114  VIF=1.018 / adj R^2^=0.078 |
| Visceral fat area index, cm2/(kg/m2) | 0.001 (-0.014 to 0.016)  *p* = 0.895  VIF=1.056 / adj R^2^=0.047 | -0.002 (-0.013 to 0.009)  *p* = 0.727  VIF=1.043 / adj R^2^=0.064 | -0.005 (-0.018 to 0.009)  *p* = 0.512  VIF=1.005 / adj R^2^=0.060 | -0.006(-0.034 to 0.023)  *p* = 0.696  VIF=1.000 / adj R^2^=0.063 |
| Subcutaneous fat area index, cm^2^/(kg/m^2^) | -0.049 (-0.098 to -0.001)  *p* = 0.046  VIF=1.020/ adj R^2^=0.050 | -0.009(-0.051 to 0.033)  *p* = 078  VIF=1.014 / adj R^2^=0.064 | -0.034 (-0.095 to 0.027)  *p* = 0.271  VIF=1.002 / adj R^2^=0.061 | 0.068 (-0.084 to 0.221)  *p* = 0.376  VIF=1.011 / adj R^2^=0.067 |

Abbreviation: PTA, pure-tone averages of 1kHz, 2kHz, 3kHz, 4kHz thresholds; Adj R^2^, adjusted R^2^
